# Species–area relationships in the Andaman and Nicobar Islands emerge because rarer species are disproportionately favored on larger islands

**DOI:** 10.1002/ece3.6480

**Published:** 2020-07-01

**Authors:** Leana D. Gooriah, Priya Davidar, Jonathan M. Chase

**Affiliations:** ^1^ German Centre for Integrative Biodiversity Research (iDiv) Halle‐Jena‐Leipzig Leipzig Germany; ^2^ Department of Ecology & Environmental Sciences Pondicherry University Pondicherry India; ^3^ Sigur Nature Trust Nilgiris India; ^4^ Institute for Computer Science Martin Luther University Halle‐Wittenberg Halle Germany

**Keywords:** alpha diversity, beta diversity, disproportionate effects, gamma diversity, heterogeneity, individual‐based rarefaction, island biogeography, passive sampling, species–area relationship

## Abstract

The island species–area relationship (ISAR) describes how the number of species increases with increasing size of an island (or island‐like habitat), and is of fundamental importance in island biogeography and conservation. Here, we use a framework based on individual‐based rarefaction to infer whether ISARs result from passive sampling, or whether some processes are acting beyond sampling (e.g., disproportionate effects and/or habitat heterogeneity). Using data on total and relative abundances of four taxa (birds, butterflies, amphibians, and reptiles) from multiple islands in the Andaman and Nicobar archipelago, we examine how different metrics of biodiversity (total species richness, rarefied species richness, and abundance‐weighted effective numbers of species emphasizing common species) vary with island area. Total species richness increased for all taxa, as did rarefied species richness controlling for a given sampling effort. This indicates that the ISAR did not result because of passive sampling, but that instead, some species were disproportionately favored on larger islands. For birds, frogs, and lizards, this disproportionate effect was only associated with species that were rarer in the samples, but for butterflies, both more common and rarer species were affected. Furthermore, for the two taxa for which we had plot‐level data (reptiles and amphibians), within‐island β‐diversity did not increase with island size, suggesting that within‐island compositional effects were unlikely to be driving these ISARs. Overall, our results indicate that the ISARs of these taxa are most likely driven by disproportionate effects, that is, where larger islands are important sources of biodiversity beyond a simple sampling expectation, especially through their influence on rarer species, thus emphasizing their role in the preservation and conservation of species.

## Graphical abstract

Here, we show that the island species–area relationships (ISARs) of birds, butterflies, amphibians, and reptiles in the Andaman and Nicobar islands are a result of processes beyond random sampling, where species are disproportionately favored on larger islands. Using an abundance‐weighted diversity measure, we show that this disproportionate effect is primarily due to changes in the abundance of rare species. Overall, our results emphasize the importance of larger islands as sources of rare species.

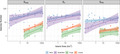

## 1 INTRODUCTION

The island species–area relationship (ISAR) describes the relationship between the number of species on an island and the area of that island, and has served as a basis for some of the most important theories in biodiversity studies (MacArthur & Wilson, 1967; Warren et al., 2015). While the general pattern and shape of the ISAR are generally positive (e.g., Matthews et al., 2016; Triantis, Guilhaumon, & Whittaker, 2012), there remains uncertainty about the mechanisms underlying the ISAR and how they shape it (e.g., Chase et al., 2019). A deeper understanding of these mechanisms will not only provide insight into the processes that shape biodiversity and its variation on islands, but will also be important for devising plans for conserving biodiversity on islands, which house a disproportionate amount of diversity compared with their land area, but are also disproportionately influenced by human impacts and global change (Tershy, Shen, Newton, Holmes, & Croll, 2015; Vitousek et al., 1997).

The simplest explanation leading to the positive ISAR is passive sampling. With passive sampling, larger islands have more individuals and as a result, a higher likelihood of sampling more species from the regional pool than smaller islands (Connor & McCoy, 1979). Coleman (1981) provided an analytical approach to evaluate this null model, which Coleman, Mares, Willig, and Hsieh (1982) subsequently tested with bird abundances on islands, finding that they could not reject the null hypothesis of passive sampling. Indeed, when appropriate data were available, passive sampling has been implicated in a number of empirical studies of ISAR patterns (e.g., Bidwell, Green, & Clark, 2014; Gooriah & Chase, 2020; Haila, 1983; Hill, Curran, & Foody, 1994; Ouin et al., 2006). Other times, however, studies have rejected the passive sampling hypothesis (e.g., Bolger, Alberts, & Soule, 1991; Ranta & As, 1982; Schoereder et al., 2004; Wang, Bao, Yu, Xu, & Ding, 2010; Xu, Han, Zhang, Millien, & Wang, 2017).

If the passive sampling hypothesis is rejected, two classes of biological mechanisms can be invoked. First, island size can disproportionately influence some species relative to others (when passive sampling is operating, effects are proportional); Connor and McCoy (1979) called these “*area* per se*”* effects to indicate that island area itself influences the relative abundances and likelihood of co‐occurrence among species, and Chase et al. (2019) more generally called these “disproportionate” effects. One prominent mechanism leading to disproportionate effects is the colonization–extinction dynamics inherent to the equilibrium theory of island biogeography, whereby extinction rates are higher and/or colonization rates are lower on smaller islands (MacArthur & Wilson, 1963, 1967). Likewise, population‐level demographic processes such as Allee effects or demographic stochasticity may tend to be more pronounced on smaller rather than larger islands, which can also lead to disproportionate effects.

Second, an increase in habitat heterogeneity with island area can also lead to more species on bigger islands (Kohn & Walsh, 1994), particularly when species require specific or multiple habitat types (Guadagnin & Maltchik, 2007; Hart & Horwitz, 1991; Williams, 1964). However, disentangling disproportionate effects from habitat diversity can prove to be quite challenging as they can easily be confounded (Boecklen & Gotelli, 1984; Connor & McCoy, 1979; Gilbert, 1980; Kohn & Walsh, 1994); that is, bigger islands tend to have more diverse habitats (Hortal, Triantis, Meiri, Thébault, & Sfenthourakis, 2009). Furthermore, it is possible that area and habitat diversity together can better explain the variation of species patterns across islands (Davidar, Yoganand, & Ganesh, 2001; Ricklefs & Lovette, 1999; Triantis, Mylonas, Lika, & Vardinoyannis, 2003). Even within the same island archipelago, it is possible that different mechanisms underlie the response of different taxa to island area. For example, in a study of the ISAR of Caribbean islands, Ricklefs and Lovette (1999) suggested that birds were more likely responding to area alone, while habitat diversity effects were likely to be stronger for butterflies, amphibians, and reptiles, and speculated that this difference might have been, at least in part, due to differences in dispersal capacity.

In this study, we evaluated the null hypothesis of passive sampling versus disproportionate and habitat heterogeneity effects leading to the ISAR of four taxa that differ in their dispersal capacity—birds, butterflies, frogs, and lizards—from the Andaman Islands in the Bay of Bengal. We used a recently developed set of analytical tools based on individual‐based rarefaction and extrapolation techniques (Chase et al., 2019). For each taxon, we used previously collected data to explicitly test the null hypothesis of passive sampling against ecological mechanisms underlying the ISAR; for frogs and lizards, we additionally were able to use spatially explicit plot‐level data, which allowed us to test the potential role of habitat heterogeneity.

## 2 MATERIAL AND METHODS

### 2.1 Study site and sampling methods

**FIGURE 1:**
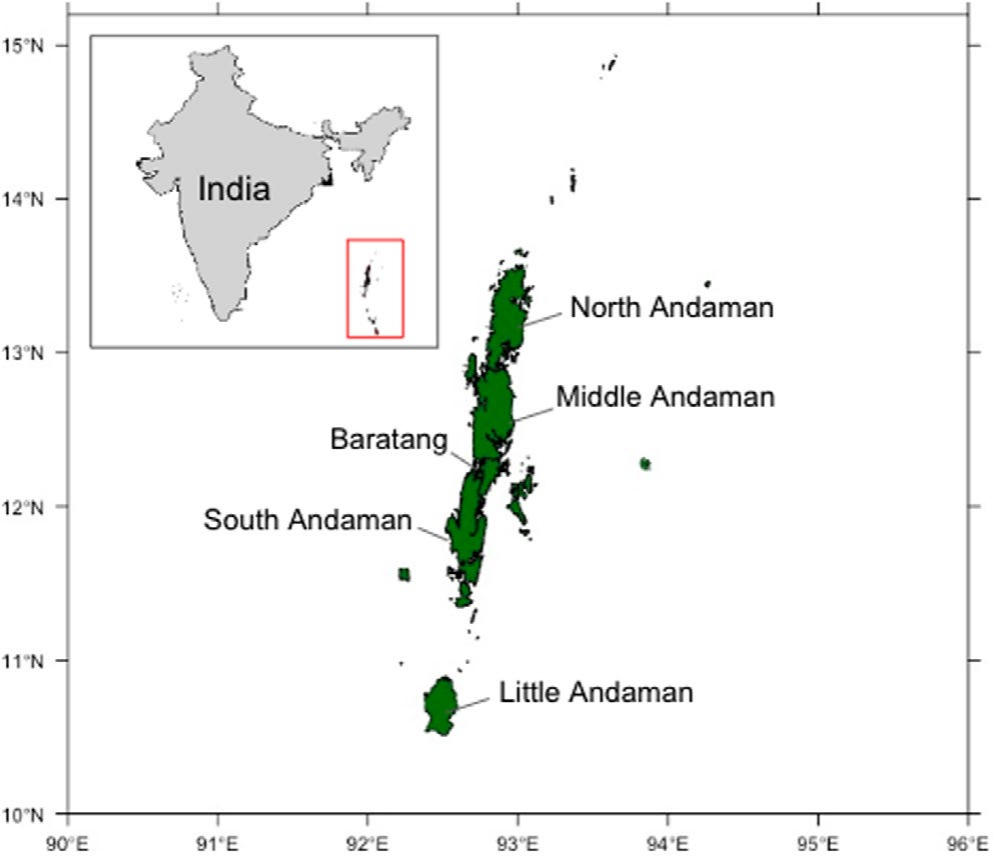
Map of the Andaman Islands, with the four main regions being North, Middle, Baratang, and South Andaman

The Andaman archipelago includes 556 islands, islets, and rocks and is made up of four large contiguous regions: North, Middle, Baratang, and South Andaman forming over 5,000 km^2^ in total area, surrounded by many isolated islands (Figure 1). The forest types across islands are diverse, ranging from evergreen forests to deciduous forests and mangroves (Champion & Seth, 1968; Davidar, Yoganand, Ganesh, & Devy, 2002). Below, we describe the sampling methodology in more detail with specific respect to the analyses we perform here. For more details on the methods and original motivation for data collection, we refer the reader to Davidar, Yoganand, Ganesh, and Joshi. (1996) and Devy, Ganesh, and Davidar (1998) for birds and butterflies, and to Surendran and Vasudevan (2015) for data collection of the frogs and lizards.

Bird surveys were carried out on 38 of the islands varying in size from 0.03 to 1,375 km^2^ as part of the study by Davidar et al. (1996). Data on the total abundances of species identified from these surveys were previously unpublished. Forest‐dwelling birds on each island were sampled between 1992 and 1994 during the dry seasons, along 1 km length transects. Transects were sampled starting at 07:00 hr, and all birds heard or seen along that transect were recorded. Each transect on each island was sampled once. Islands contain multiple forest types and the number of habitat types typically increases with island area (Davidar et al., 2001). Habitat types were classified into three groups: littoral forests, deciduous forests, and wet forests (i.e., evergreen or semievergreen forest). On smaller islands, transects typically cut through multiple habitats on those islands and were thus sampled roughly in proportion to their availability on that island. On larger islands, transects were placed within each habitat type and the number of transects per habitat type was varied roughly in proportion to the total amount of that habitat on each island. Unfortunately, metadata on the numbers of individuals surveyed from each transect/habitat type combination were not retained from the original surveys, and the abundances of each species were pooled across each island. Thus, we do not detail further information on habitat types or the spatial differences among transects, nor do we explicitly quantify habitat diversity. Instead, we simply take these surveys as representative surveys of each island bird community, including relative abundances, given its level of habitat diversity (which is known to increase with island area; Davidar et al., 2001). With these data, even with unequal sampling efforts, the rarefaction and extrapolation methods that we use to analyze these data allow us to ask whether observed diversity and relative abundances of species differed from what would have been expected from passive sampling alone (see Chase et al., 2019 and Section 2.2 below). In total, 5,532 individual birds of 47 species were detected across the 38 sampled islands.

Butterfly surveys were also performed from 1992 to 1994 during the dry seasons on 25 islands that ranged from 0.03 to 1,350 km^2^ (for more details on survey methodology, see Devy et al., 1998). On these islands, observers walked transects and identified all butterflies seen to species (or nearest morphospecies) within 5 m of the transect. Surveys took place between 08:00 and 12:00 hr. On small islands, observers walked serpentine across the entire island and recorded all individuals seen. On large‐ and medium‐sized islands, observers walked variable‐length transects across the entire island. Surveys were continued over multiple days until several transects were run with no new species observed. As above, surveys were conducted roughly in proportion to habitat type availability, which included evergreen, disturbed evergreen, deciduous, disturbed deciduous, littoral, and edge habitat. However, of the 25 islands surveyed, 17 had incomplete abundance data (i.e., the numbers of individuals were not reported), and therefore, we did not include these islands in our analysis. As with birds, metadata were not retained, and individuals were pooled across all transects on each island. We could then use these pooled communities with the rarefaction and extrapolation methods to ask whether observed patterns differed from that expected from passive sampling (see Chase et al., 2019 and Section 2.2 below). In all, observers found 982 individual butterflies of 51 species across 8 islands.

Frog and lizard surveys using open and bounded quadrats were carried out on 15 islands between 2010 and 2012 and were previously published by Surendran and Vasudevan (2015). Here, we analyze the data from surveys carried out on the 14 islands (varying in size from 0.09 to 1,375 km^2^) that only used bounded quadrats (10 m × 10 m) during the dry seasons (November to May) of 2010–2011 and 2011–2012 (for more details, see Surendran & Vasudevan, 2015). Quadrats were placed in evergreen forest types, which are the predominant habitat on these islands, on relatively flat terrain. Thus, the authors explicitly intended to minimize habitat heterogeneity effects on diversity estimates across islands that varied in size. The number of quadrats sampled increased with increasing island size, ranging from two to ten quadrats (we did not include data from the two islands where only one quadrat was surveyed in our analysis). Here, information on the numbers of individuals within each plot was retained and reported, allowing us to calculate patterns of smaller and larger‐scale diversity on each island. Of the 12 islands surveyed (with more than two quadrats), one had no lizards and two had no frogs, and these were eliminated from their respective analyses. Therefore, in total, 754 individual lizards of 9 species were detected across 11 islands, and 302 individual frogs of 8 species were detected across 10 islands.

### 2.2 Hypotheses and analyses

To untangle the potential mechanisms underlying the ISAR for these groups, we follow the framework for questions, hypotheses, and analyses outlined in Chase et al. (2019). Analyses and calculations are described in more detail below, and R code specific for these analyses is available on GitHub https://github.com/LeanaGooriah/ISAR_analysis.

#### 2.2.1 Is there a relationship between island area and the total number of species on an island?

We did not have independent estimates of the total number of species on each island (which we refer to as *S*_total_), and thus, we used an extrapolation technique using the survey data to establish whether there was an overall ISAR for each group. Specifically, we estimated *S*_total_ from each island by combining abundance data from all plots and using the Chao1 estimator to estimate the number of unseen species from each island (Chao, 1984; Hsieh, Ma, & Chao, 2016), calculated as follows:

$$S_{1}=S_{\text{obs}}+\frac{F_{1}^{2}}{2F_{2}}$$

where *S*_obs_ is the number of species in the sample, *F*_1_ is the number of singletons, and *F*_2_ is the number of doubletons. This value should be taken as a minimum possible number of species on each island. The Chao1 estimator is part of a family of measures that can be derived from the individual‐based rarefaction curve (e.g., Chao & Jost, 2012; Chase et al., 2019; Colwell et al., 2012); specifically, this measure estimates the asymptote of the rarefaction curve based on the available sample data (i.e., extrapolation to full coverage) and thus estimates the total number of species on a given island (*S*_total_). It should be clearly noted, however, that (a) the Chao1 estimator can only be a lower estimate of expected richness, and (b) the main objective of our study is not on establishing whether there is a relationship between total richness and island size, which has been shown many times previously, but rather to examine the sample‐effort controlled patterns of species richness that can be examined with rarefaction. That is, simply establishing a relationship between *S*_total_ and island area does not allow us to go further into dissecting the possible mechanisms underlying the ISAR relationship.

#### 2.2.2 Can we reject the null hypothesis of passive sampling?

**FIGURE 2:**
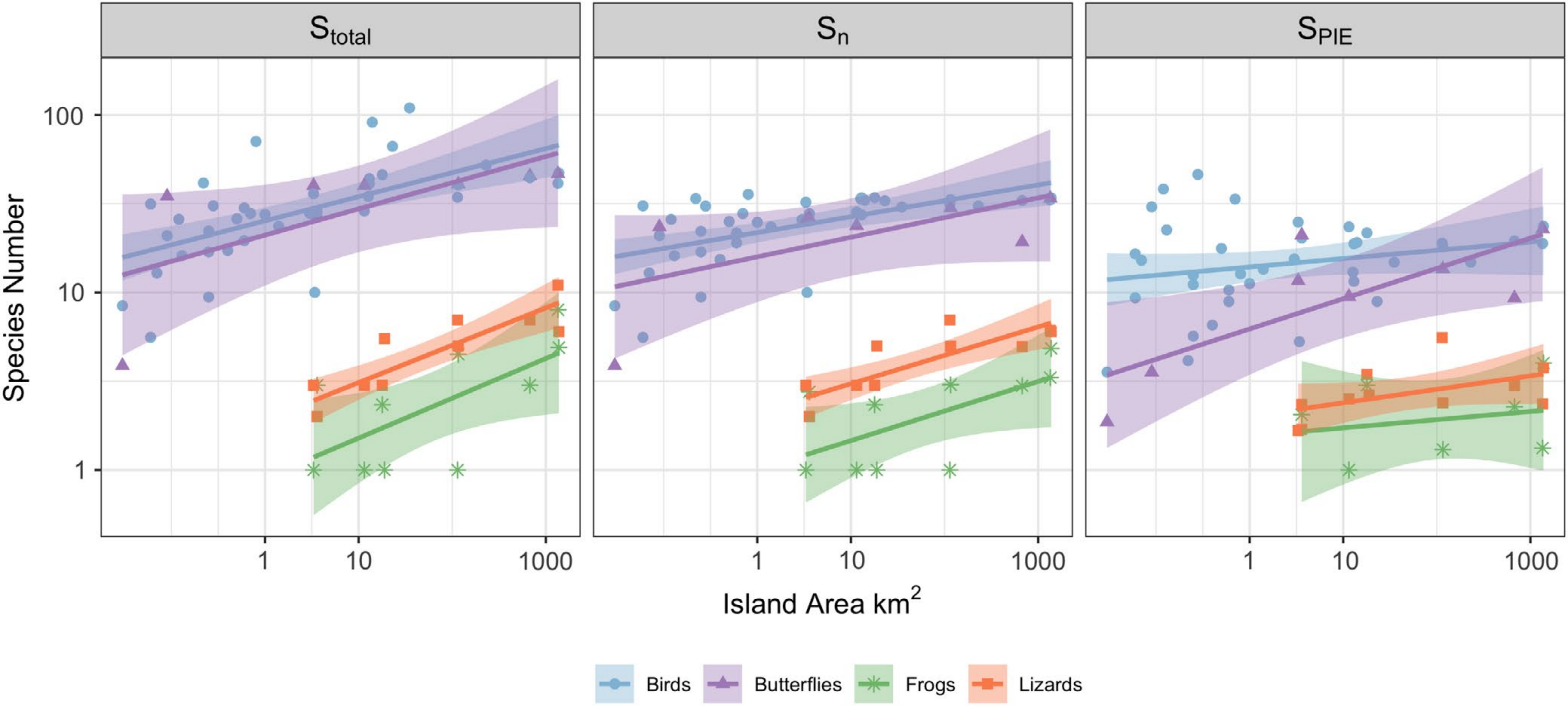
Linear regressions of log‐transformed biodiversity metrics against the log of island area (km2) for all four taxa. Variables include the total number of species estimated per island from the pooled abundance data (Stotal), the number of species expected at a specific number of individuals (Sn) (where the numbers of species are rarefied to a reference n, nref) and the corresponding effective number of species of the probability of interspecific encounter (SPIE)

We used individual‐based rarefaction to evaluate whether the ISAR results deviate from passive sampling, or if instead, some biological mechanism can be invoked. This approach, similar to the random placement model of Coleman (1981), uses the individual‐based rarefaction curve calculated from all of the transects/quadrats taken from each island. From each individual‐based rarefaction curve per island, we calculated the number of species expected for a given number of individuals (i.e., *n* individuals that are randomly drawn from each island), which we term *S*_n_ (Figure 2). In this case, we rarefied species richness to a reference *n*, which is a base sample size that we used for extrapolation and interpolation of the rarefaction curve. Following the recommendations of Chao et al., 2014, we calculated this reference *n,* called *n*_ref_, by:

1. Calculating the maximum reference sample size, *n*_max_, which is the maximum number of individuals on an island.
2. Doubling the minimum sample size, *n*_min_ (i.e., the minimum number of individuals recorded across all transects/quadrats), that is, *n_b_ *= *n*_min_ * 2. Here, *n_b_* would be the minimum base sample size.
3. Our reference *n*, *n*_ref_, would be the maximum value of the two values *n*_max_ and *n*_b_.

$$n_{\text{ref}}=\max\left[n_{\max},n_{b}\right].$$

If there is no relationship between *S_n_* and island size, then we cannot reject the null hypothesis that the ISAR results from passive sampling alone. Alternatively, if *S_n_* increases with island size, we can conclude that there is some other mechanism operating that allows more species to co‐occur for a given n on larger than smaller islands. This allows us to reject the null hypothesis of passive sampling and indicates that disproportionate effects and/or heterogeneity are playing a role in driving the patterns.

To discern whether any changes in *S_n_* were due to changes in more common relative to rarer species in the community, we calculated a metric that is sensitive to changes in the common species, but insensitive to rarer species, Hurlbert's (1971) probability of interspecific encounter (PIE),

$$\text{PIE}=\left(\frac{N}{N-1}\right)\times \left(1-\sum_{i=1}^{S}p_{i}^{2}\right)$$

where *N* is the total number of individuals in the entire community, *S* is the total number of species in the community, and *p_i_* is the proportion of each species *i*. For analyses, we convert PIE to an effective number of species, *S*_PIE_ (Jost, 2006) (*S*_PIE_ = 1/1 − PIE and is proportional to Simpson's index; Hill, 1973, Jost, 2006). A relationship between *S*_PIE_ and island area indicates that larger islands have shifts in both common and rare species relative abundances. Alternatively, if *S*_n_ increases with island area, but *S*_PIE_ does not vary with island area, we would then conclude that only the rarer species are influenced by island area (Chase et al., 2019).

#### 2.2.3 How does within‐island β‐diversity vary with island area?

As described above, individual plot‐level data were not available for the surveys on birds and butterflies, and so our analyses above are relevant to patterns at the island (not individual plot) scale. However, we did have individual‐level plot data from the frog and lizard surveys, and thus, we could take our analyses a step further. We calculated within‐island β‐diversity by comparing the value of *S*_n_ calculated as the average (local) rarefied richness from a single quadrat on each island with the value of *S*_n_ when calculated from the pooled individuals across all plots (for details of these methods and their meaning, see Chase et al., 2019). The ratio of these two values can be taken as an index of β‐diversity that indicates the degree to which species are clumped in the landscape thus distributed heterogeneously across an island. The same can be done for *S*_PIE_ to determine whether the clumping is due to more common or rare species (see also Olszewski, 2004). If there is no relationship between either of these β‐diversity metrics and island size, we can reject the hypothesis that the ISAR results from within‐island compositional variation, whereas if measures of β‐diversity increase with island size, we can conclude that compositional variation plays a role underlying the ISAR (Chase et al., 2019; Kallimanis et al., 2008).

### 2.3 Statistical analysis

We calculated total estimated species richness (*S*_total_), the rarefied number of species expected for a given number of individuals (*S*_n_), and the effective number of species (*S*_PIE_) using the R package mobr (McGlinn et al., 2019). Additionally, for lizards and frogs, we calculated these from the pooled data across each island, as well as the plot‐level data in order to derive β‐diversity indices. Code specific for ISAR analyses is available on GitHub https://github.com/LeanaGooriah/ISAR_analysis. For each taxon, we used linear regression to evaluate the relationship between log‐transformed diversity indices (*S*_total_, *S*_n_, *S*_PIE_) and the log of island size.

## 3 RESULTS

Figure 2 illustrates the ISAR relationship for each taxon for each diversity measure and Table 1 gives the regression coefficients. For all four taxa, total species richness (*S*_total_) increased with island size, indicating a positive overall ISAR. Likewise, when calculated from the entire list of species and their relative abundances, rarefied species richness, *S*_n_, increased with island size. This positive relationship between *S*_n_ and island size allows us to reject the null hypothesis of passive sampling for each taxon, as it would be expected to be constant with island size under passive sampling. For birds, lizards and frogs, we found no concomitant increase of *S*_PIE_ with island size, suggesting the disproportionate effect was most strongly affecting the presence of less common species. For butterflies, however, *S*_PIE_ also increased with increasing island size, suggesting that communities became more even on larger islands.

**TABLE 1:** Regression models and their estimates of intercept, slope, and *R* ^2^

Taxa	Response	logC ± *SE*	*z* ± *SE*	*R* ^2^	*p*‐value
Birds	**log *S*_total_ ~ log Area**	**3.23 ± 0.08**	**0.14 ± 0.03**	**.38**	**<.0001**
	**log *S*_n_ ~ log Area**	**3.07 ± 0.06**	**0.08 ± 0.02**	**.31**	**.0001**
	log *S*_PIE_ ~ log Area	2.63 ± 0.10	0.05 ± 0.03	.03	.15
Butterflies	**log *S*_total_ ~ log Area**	**3.04 ± 0.27**	**0.14 ± 0.06**	**.39**	**.05**
	**log *S*_n_ ~ log Area**	**2.76 ± 0.23**	**0.11 ± 0.05**	**.29**	**.09**
	**log *S*_PIE_ ~ log Area**	**1.82 ± 0.24**	**0.17 ± 0.05**	**.53**	**.02**
Lizards	**log *S*_total_ ~ log Area**	**0.64 ± 0.16**	**0.21 ± 0.03**	**.77**	**.0002**
	**log *S*_n_ ~ log Area**	**0.75 ± 0.15**	**0.16 ± 0.03**	**.67**	**.001**
	log *S*_PIE_ ~ log Area	0.70 ± 0.18	0.07 ± 0.04	.18	.10
Frogs	**log *S*_total_ ~ log Area**	**−0.10 ± 0.41**	**0.22 ± 0.08**	**.37**	**.03**
	**log *S_n_* ~ log Area**	**−0.002 ± 0.34**	**0.16 ± 0.07**	**.31**	**.05**
	log *S*_PIE_ ~ log Area	0.44 ± 0.45	0.046 ± 0.08	−.13	.62

Bold indicates significant values where *p*‐value ≤.05.

**FIGURE 3:**
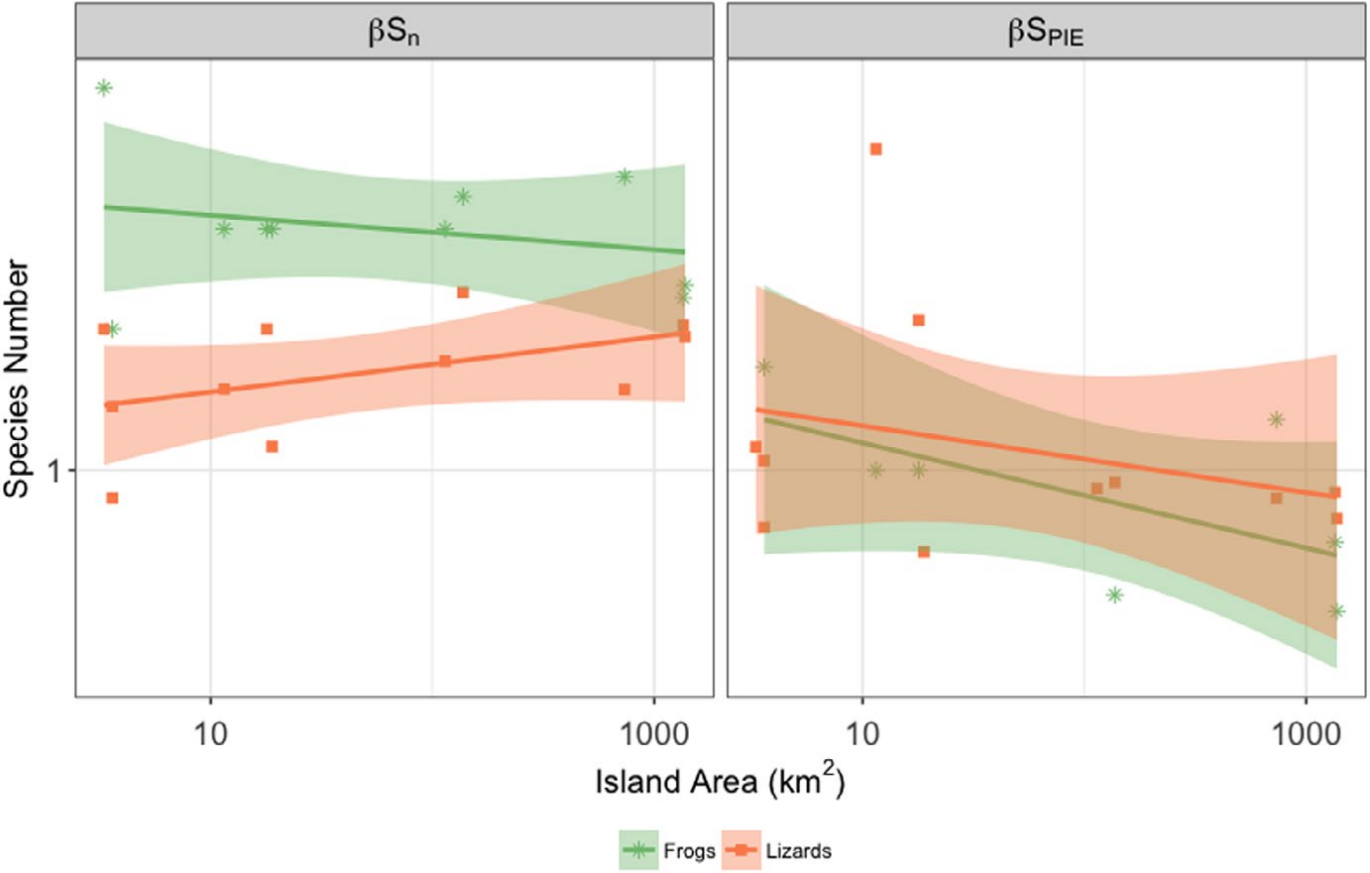
Linear regressions of log‐transformed variables Sn and SPIE at the β‐scale against the log of island area (km^2^) for frogs and lizards

We estimated β‐diversity for *S*_n_ and *S*_PIE_ for frogs and lizards because we had plot‐level data available and then regressed log‐transformed values against the log of island area. We found no influence of island size on either of the β‐diversity measures (Figure 3, for all four linear regressions: *p* > .1).

## 4 DISCUSSION

Overall, we found that island size had a positive significant effect on estimated species richness of bird, butterfly, frog, and lizards at the whole island scale (*S*_total_) among islands that varied in size in the Andaman archipelago. This result is expected from both theory and frequent observation (e.g., Connor & McCoy, 1979; MacArthur & Wilson, 1967; Triantis et al., 2012). However, less attention has been paid toward dissecting the potential mechanisms underlying the ISAR, in particular, differentiating the passive sampling hypothesis from ecological mechanisms leading to disproportionate effects (e.g., Chase et al., 2019; Coleman et al., 1982; Hill et al., 1994). Here, we used a rarefaction‐based analytical approach to compare the mechanisms underlying the ISAR among four animal taxa that vary in life history and dispersal characteristics. Despite their inherent biological differences, we found a generally consistent pattern that the island‐wide rarefied species richness (*S*_n_) increased with island size for all four taxa. This means that for each taxon, regardless of life history and dispersal mode, among other biological differences, more species persist for a given number of individuals than would be expected from passive sampling alone and that processes beyond sampling are operating in the ISAR.

We used the effective number of species estimated from a relatively unbiased evenness metric, *S*_PIE_, which is relatively insensitive to rare species, to discern the influence of island size on the overall shape of the species abundance distribution. If *S*_n_ varies with island area, but not *S*_PIE_, then only the rarer species from samples are more likely to be found on larger islands than from a passive sampling expectation. This is exactly what we found for birds, frogs, and lizards. This could have emerged, for example, because populations on larger islands are more likely to persist by avoiding Allee effects and/or demographic stochasticity (Hanski & Gyllenberg, 1993; Orrock & Watling, 2010), or through the increased likelihood of specialized habitats on larger islands (Davidar et al., 2001; Kohn & Walsh, 1994; Williams, 1964). Importantly, we note that because there were only 10 and 11 islands in the frog and lizard analyses, respectively, the lack of a relationship between *S*_PIE_ and island size should be taken with caution.

For butterflies, we found that both *S*_n_ and *S*_PIE_ increased with increasing island area, suggesting that the entire shape of the relative abundance distribution became more even on larger islands. Without further information, we cannot explicitly test why butterflies might have differed in their responses to island size compared with the other taxa. However, we might speculate that due to their larger population sizes and higher levels of specialization (especially in the larval stage), butterflies are poised to have altered relative abundance distributions on larger islands with higher plant and habitat diversity.

Because we had plot‐level information available, we were able to compare within‐island β‐diversity measures for frogs and lizards. Here, we found that even though larger islands in this archipelago do have more heterogeneity in habitat types and have a higher proportion of wet evergreen forests that support rarer species (Davidar et al., 2001; Yoganand & Davidar, 2000), this did not result in an influence of island size on β‐diversity of these two taxa despite the fact that they are relatively poor dispersers (Cook & Quinn, 1995; Quinn & Harrison, 1988; Watling & Donnelly, 2006). However, we note two limitations of our β‐diversity analysis that should caution overinterpretation of this negative result. First, there were only 10 and 11 islands for frogs and lizards respectively in this analysis, and so the power of the regressions was necessarily weak. Second, the data collection by Surendran and Vasudevan (2015) was focused primarily within a single habitat type, intentionally minimizing variation in habitats among islands. Thus, any variation in β‐diversity among islands that we could have observed here would have been primarily a result of spatial variation in community composition that emerged from factors other than habitat heterogeneity. Nevertheless, with the data we have in hand, we conclude that the deviations from the passive sampling hypothesis, at least for these two taxa, were more likely to have resulted from an ecological mechanism other than habitat heterogeneity and/or within‐island dispersal limitation. Increased habitat heterogeneity on larger islands could also play a role in addition to the effects we observed here. To be able to more fully evaluate this, one would need to compare observed within‐island β‐diversity patterns with variation in local environmental conditions in a more spatially explicit way than was possible with the datasets we had available to us here.

While our results point to a strong influence of island size on both the total number of species and the numbers of species when sample effort was controlled with rarefaction, we cannot exclude other variables influencing the species diversity relationships other than area. For example, in a study involving plants on small islands, Panitsa, Tzanoudakis, Triantis, and Sfenthourakis (2006) found strong island species–area relationships but factors such as elevation and the presence of grazing species also explained some of the variance. Another important variable influencing island species–area relationships is isolation, such as distance among islands or to the mainland (Kreft, Jetz, Mutke, Kier, & Barthlott, 2007; MacArthur & Wilson, 1967). Isolation could further be quantified by accounting for islands that act as stepping stones and the size of neighboring land masses, where large islands act as important colonization sources (Weigelt & Kreft, 2013). Nevertheless, most of the islands included in our analysis and the Andaman island group in general are quite close to one another and the mainland is made up of four main contiguous regions, so isolation may not have been a likely contributing factor in this case.

In conclusion, our study is relatively unique in that we explicitly examined the potential mechanisms underlying positive ISARs for multiple taxa that vary in a number of ecological characteristics within a single island archipelago. For each group, birds, butterflies, frogs, and amphibians, we could reject the null hypothesis of passive sampling to indicate that the ISAR likely resulted from some underlying ecological mechanism beyond sampling. While habitat heterogeneity certainly plays a role, this was not likely to be the sole reason for the positive effects on larger islands. Thus, larger islands are important sources of biodiversity beyond a simple sampling expectation, especially through their influence on rarer species. This is especially important in nature conservation and planning since smaller islands are often given higher priority mainly when establishing nature reserves (Davidar, Devy, Yoganand, & Ganesh, 1995). The protection and presence of nature reserves on larger islands could therefore be an effective way of conserving species.

## CONFLICT OF INTEREST

The authors declare that they have no conflict of interest.

## AUTHOR CONTRIBUTION

**Leana D. Gooriah:** Conceptualization (equal); Data curation (lead); Formal analysis (lead); Methodology (equal); Writing‐original draft (lead); Writing‐review & editing (equal). **Priya Davidar:** Conceptualization (supporting); Data curation (supporting); Formal analysis (supporting); Methodology (supporting); Writing‐original draft (supporting); Writing‐review & editing (supporting). **Jonathan M. Chase:** Conceptualization (equal); Data curation (supporting); Formal analysis (supporting); Methodology (equal); Writing‐original draft (supporting); Writing‐review & editing (equal).

## Data Availability

The data that support the findings of this study are openly available in the Dryad Digital Repository at https://doi.org/10.5061/dryad.7m0cfxpr5.
